# Expression of Calgranulin Genes S100A8, S100A9 and S100A12 Is Modulated by n-3 PUFA during Inflammation in Adipose Tissue and Mononuclear Cells

**DOI:** 10.1371/journal.pone.0169614

**Published:** 2017-01-26

**Authors:** Rachana D. Shah, Chenyi Xue, Hanrui Zhang, Sony Tuteja, Mingyao Li, Muredach P. Reilly, Jane F. Ferguson

**Affiliations:** 1 Division of Pediatric Endocrinology, Children’s Hospital of Philadelphia, Pennsylvania, United States of America; 2 Department of Medicine, Perelman School of Medicine at the University of Pennsylvania, Philadelphia, Pennsylvania, United States of America; 3 Department of Medicine, Columbia University, New York, New York, United States of America; 4 Department of Biostatistics & Epidemiology, University of Pennsylvania, Philadelphia, Pennsylvania, United States of America; 5 Division of Cardiovascular Medicine, and Vanderbilt Translational and Clinical Cardiovascular Research Center (VTRACC), Vanderbilt University Medical Center, Nashville, Tennessee, United States of America; Chiba University Graduate School of Medicine, JAPAN

## Abstract

Calgranulin genes (*S100A8*, *S100A9* and *S100A12*) play key immune response roles in inflammatory disorders, including cardiovascular disease. Long-chain omega-3 polyunsaturated fatty acids (LC n-3 PUFA) may have systemic and adipose tissue-specific anti-inflammatory and cardio-protective action. Interactions between calgranulins and the unsaturated fatty acid arachidonic acid (AA) have been reported, yet little is known about the relationship between calgranulins and the LC n-3 PUFA eicosapentaenoic acid (EPA) and docosahexaenoic acid (DHA). We explored tissue-specific action of calgranulins in the setting of evoked endotoxemia and n-3 PUFA supplementation. Expression of calgranulins in adipose tissue *in vivo* was assessed by RNA sequencing (RNASeq) before and after n-3 PUFA supplementation and evoked endotoxemia in the fenofibrate and omega-3 fatty acid modulation of endotoxemia (FFAME) Study. Subjects received n-3 PUFA (n = 8; 3600mg/day EPA/DHA) or matched placebo (n = 6) for 6–8 weeks, before completing an endotoxin challenge (LPS 0.6 ng/kg). Calgranulin genes were up-regulated post-LPS, with greater increase in n-3 PUFA (*S100A8* 15-fold, p = 0.003; *S100A9* 7-fold, p = 0.003; *S100A12* 28-fold, p = 0.01) compared to placebo (*S100A8* 2-fold, p = 0.01; *S100A9* 1.4-fold, p = 0.4; *S100A12* 5-fold, p = 0.06). In an independent evoked endotoxemia study, calgranulin gene expression correlated with the systemic inflammatory response. Through *in vivo* and *in vitro* interrogation we highlight differential responses in adipocytes and mononuclear cells during inflammation, with n-3 PUFA leading to increased calgranulin expression in adipose, but decreased expression in circulating cells. In conclusion, we present a novel relationship between n-3 PUFA anti-inflammatory action *in vivo* and cell-specific modulation of calgranulin expression during innate immune activation.

## Introduction

Calgranulin genes, which include *S100A8*, *S100A9* and *S100A12*, are members of the S100 gene family of calcium binding proteins. These proteins have known functions in immune response, and act as damage-associated molecular pattern (DAMP) proteins, signaling through the receptor for advanced glycation end products (RAGE), and acting as endogenous toll-like receptor 4 (TLR4) ligands.[1, 2] These genes are particularly abundant in myeloid cells, with high protein levels within the cytoplasm of neutrophils and monocytes.[3] *S100A12* preferentially forms a homodimer,[4] and may form a tetramer or hexamer,[5] while *S100A8* and *S100A9* (also known as *MRP8* and *MRP14*) form calprotectin, present as a heterodimer or heterotetramer; [6, 7] *S100A8* and *S100A9* may also form homodimers and other higher complexes, subject to post-translational modification[8], with distinct functionality from calprotectin.

The specific function and disease relevance of calgranulin genes is complex. Expression and protein levels are increased during immune activation and inflammation, in diverse disease settings including diabetes, obesity, cardiovascular disease, arthritis, cancer, pancreatitis, psoriasis, inflammatory bowel disease, and pathogenic infection.[9–14] Serum S100A12 associates with risk of CAD in subjects with T2DM.[15] However, whether calgranulins play a significant regulatory role in disease or are useful as cardiovascular disease markers is not yet clear.[9, 16] S100A8 and A9 recruit macrophages to adipose tissue from bone marrow myeloid progenitors during inflammation and in the setting of hyperglycemia and obesity,[17–19] and may regulate inflammation through inflammasome activation.[20] S100A8 and A9 are thought to have both pro- and anti-inflammatory action, acting in a cell-type specific manner[21], with potential importance in the resolution of inflammation.[22, 23] They have distinct intracellular and extracellular functions, potentially mediated by differences in Ca^2+^ concentrations within the cell compared with the extracellular environment, and may exert anti-microbial action through sequestration of metal ions, thus depriving pathogens of essential nutrients.[13] As a heterodimer, but not as individual subunits, S100A8/A9 may function as a fatty acid carrier in neutrophils by forming a complex with selected fatty acids, preferentially binding unsaturated fatty acids.[24, 25] S100A8/A9 preferentially binds endothelial cells *in vitro* in the presence of the n-6 polyunsaturated fatty acid arachidonic acid when compared with saturated and monounsaturated fatty acids.[26] S100A8/A9 stability has been reported to be altered in the presence of unsaturated fatty acids, and thus the specific fatty acid milieu may alter S100A8/A9 functionality during inflammation.[27] However, to our knowledge, calgranulin modulation and interaction with long-chain n-3 PUFA has not been studied.

We examined calgranulin gene expression in adipose tissue after n-3 PUFA supplementation and *in vivo* lipopolysaccharide (LPS) challenge in the Fenofibrate and omega-3 fatty acid modulation of endotoxemia (FFAME) Study.[28] We found significant up-regulation of *S100A8*, *S100A9* and *S100A12* in response to LPS *in vivo*, with evidence of markedly enhanced induction in the setting of high-dose (3600mg/day EPA/DHA) supplemental n-3 PUFA. In an independent human endotoxemia study (GENE), we found a tissue-specific relationship between calgranulin expression and the systemic inflammatory response, with higher adipose tissue expression associated with lower systemic inflammation, while higher expression in circulating mononuclear cells is associated with increased systemic inflammation. Finally, we provide integrative genomic and *in vitro* evidence for these genes as cell-specific, novel nutrient-dependent regulators of inflammatory responsiveness.

## Materials and Methods

### The FFAME Study

As described,[28] the FFAME Study (clinicaltrials.gov, NCT01048502) recruited healthy volunteers (N = 60, 43% Female, 65% European ancestry [EA], 20% African ancestry [AA], 15% Asian ancestry) to the University of Pennsylvania (UPenn) for a placebo-controlled (corn oil (99.4%) with α-tocopherol (0.6%) as an antioxidant). 6–8 week n-3 PUFA supplementation trial (EPA/DHA at 900mg/day or 3600mg/day) followed by an inpatient evoked endotoxemia protocol (0.6ng/kg LPS). Samples of gluteal subcutaneous adipose tissue were obtained and snap-frozen for subsequent RNA extraction. A subset of EA subjects (n = 6 placebo, n = 8 n-3 PUFA 3600mg/day; 50% Female) were selected for adipose tissue RNA sequencing (RNASeq) of adipose biopsies obtained at baseline, after supplementation, and after LPS challenge (4hrs post-LPS)[29]. The trial was conducted with the approval of the UPenn Institutional Review Board, and all participants provided written informed consent.

### The GENE Study

The GENE Study recruited healthy individuals (N = 294, 52% Female, 65% EA, 35% AA) ancestry to a University of Pennsylvania (UPenn) inpatient Clinical and Translational Research Center (CTRC) protocol as previously described (clinicaltrials.gov NCT00953667).[30] Participants completed an endotoxin challenge (1ng/kg *E coli*-derived LPS; U.S. standard reference, lot No. CCRE-LOT-1+2, Clinical Center, Pharmacy Department at the National Institutes of Health, Bethesda MD).[30, 31] Multiple clinical variables were assessed during the visit. Individuals were ranked by their peak clinical inflammatory response (Δ from baseline for fever, plasma TNFα, plasma IL-6). Individuals falling within the top and bottom 5% of responses were designated as “high” or “low” responders respectively. CD14+ monocytes were isolated from whole blood using magnetic bead selection (Dynabeads^®^ CD14, ThermoFisher, Waltham MA), and frozen in TRIzol reagent (ThermoFisher, Waltham MA) for subsequent RNA extraction via standard protocol.[32]. The GENE study was approved by UPenn’s Institutional Review Board (IRB), with regulatory oversight by the FDA (LPS: IND# 5984) and an NIH-appointed data-safety and monitoring board. All subjects provided written informed consent.

### Laboratory Methods

#### RNA extraction and expression analysis

RNA from FFAME Study adipose tissue biopsies (3 samples per subject) was extracted using the RNeasy total RNA kit (Qiagen Inc, Valencia, CA).[33] RNA concentration and quality was assessed using an Agilent BioAnalyzer (Agilent, Santa Clara, CA). Strand-specific poly-A RNA libraries (TruSeq RNA Sample Preparation Kit, Illumina, San Diego, CA) were prepared as described.[32] Briefly, libraries were sequenced on an Illumina HiSeq 2000 sequencer, with 6 samples per lane (~30million 2×101 bp paired-end reads per sample after filtering). RNASeq data were generated and analyzed for the GENE Study as described.[32] For the *in vitro* experiments, RNA was subjected to RT-PCR and subsequent quantitative PCR (qPCR) using pre-designed primers and probes (Applied Biosystems 7900 Real-Time PCR System, Foster City, CA) for measurement of candidate gene mRNA (*S100A8*, *S100A9*, *S100A12)*. Gene expression levels were normalized to *GAPDH* and analyzed using the relative quantitation 2^-(ΔΔCt)^ method to determine relative-change.

#### Plasma protein analysis

Blood samples were collected into EDTA tubes, mixed by inversion, and immediately placed on ice. Plasma was obtained through centrifugation, and immediately frozen and stored at -80°C for subsequent analysis. Plasma S100A9 protein was measured in a subset of individuals from the GENE study before and after LPS (n = 16) using the SOMALogic SOMAscan assay.[34] This assay panel measures 1129 proteins simultaneously using aptamer technology.

#### Cell culture experiments

For *in vitro* experiments, pre-adipocytes from the stromal vascular fraction (SVF) were isolated, cultured and differentiated to adipocytes as previously described.[35] Briefly, cells were grown to confluence in OF medium with 20% fetal bovine serum and differentiated in serum-free medium with high insulin and PPARγ agonist.[35, 36] Adipocytes were treated with EPA and DHA (50μM in dimethyl sulfoxide [DMSO]; Sigma-Aldrich, St. Louis, MO) or vehicle (DMSO) for 48 hours. THP-1 monocytes (Sigma-Aldrich, St. Louis, MO) were treated with EPA and DHA (100 μM in DMSO) or vehicle (DMSO) for 72 hours. Treatment concentration and time for each cell type was determined based on previous time and dose response experiments (data not shown). Cells were then treated with LPS (100ng/ml) for 4 hours, and harvested for RNA extraction using Trizol reagent (Life Technologies, Foster City, CA).

#### Macrophage differentiation and polarization

As described[37], peripheral blood mononuclear cells (PBMCs) were collected using Mononuclear Cell Preparation tubes (BD Vacutainer) and cultured for 7 days to induce macrophage differentiation. Macrophages were polarized using LPS and interferon-γ (M1) or IL-4 (M2)[38]. RNA was extracted (All Prep DNA/RNA/miRNA Universal Kit; Qiagen, Valencia, CA) and RNASeq libraries were prepared (TruSeq RNA Sample Preparation Kit; Illumina, San Diego, CA), and sequenced (Illumina HiSeq 2000) as described[37, 39]. RNASeq data are available from the NCBI Gene Expression Omnibus under the accession numbers GSE55536.

### Statistical Analysis and Bioinformatics

RNASeq data were aligned to the hg19 reference genome using STAR 2.3.0e[40] with default options. RNA-SeqC[41] was used to assess quality of each sample. Estimated gene expression levels from pre- to post-LPS were quantified as fragments per kilobase per million fragments mapped (FPKM), and analyzed using the cuffdiff option in Cufflinks version 2.1.1. Relative expression fold changes were calculated by dividing post-LPS by pre-LPS expression values. FFAME Study RNASeq data were deposited to Gene Expression Omnibus (GEO, http://www.ncbi.nlm.nih.gov/geo/), accession number GSE87426. GENE Study data are deposited under accession number GSE39118. Publicly available expression data (GSE5099 and GSE28070) were queried from datasets deposited in GEO and analyzed using GEO2R.[42] *In vitro* and protein data were assessed for normality of distribution, and analyzed by 1-way ANOVA, 2-way ANOVA and by Spearman correlation. Data analysis was carried out using IBM SPSS Statistics 22 and GraphPad Prism 6. All p values quoted for array-wide experiments (e.g. RNASeq, microarray, eQTL) are after adjustment for platform-specific multiple testing.

## Results

### Expression of S100A8, S100A9 and S100A12 is Increased in Adipose Tissue in Response to LPS Following n-3 PUFA Supplementation

**Fig 1:**
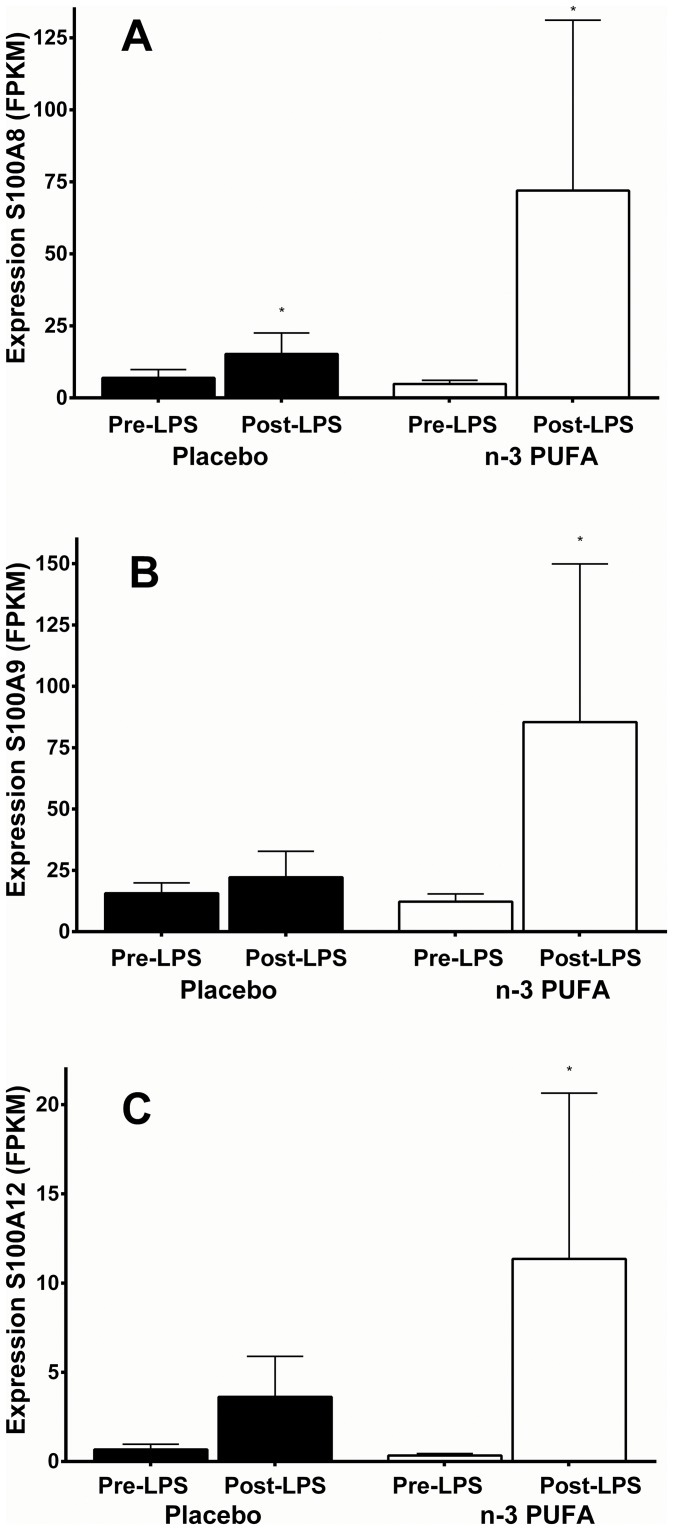
Calgranulin expression *S100A8* (A), *S100A9* (B) and *S100A12* (C) was markedly increased in adipose tissue following n-3 PUFA supplementation and LPS in FFAME Study participants (n = 6 placebo, n = 8 n-3 PUFA). * indicates p<0.01. Data plotted as mean Fragments per kilobase per million fragments mapped (FPKM) and standard error.

In the FFAME Study endotoxin challenge, *S100A8*, *S100A9* and *S100A12* expression was increased in adipose tissue post-LPS in both the placebo and the n-3 PUFA group, however this was markedly enhanced following n-3 PUFA supplementation. Expression levels of *S100A8* and *S100A9* were higher than that of *S100A12* (average pre-LPS FPKM *S100A8* = 6, *S100A9* = 14, *S100A12* = 0.5). All three calgranulin genes were highly up-regulated post-LPS in the n-3 PUFA group (*S100A8* ~15-fold, p = 0.003; *S100A9* ~7-fold, p = 0.003; *S100A12* ~28-fold, p = 0.01), while they were only moderately increased post-LPS in the placebo group (*S100A8* ~2-fold, p = 0.01; *S100A9* ~1.4-fold, p = 0.4; *S100A12* ~5-fold, p = 0.06) (Fig 1). This increased activation in the n-3 PUFA group compared with placebo reached statistical significance for *S100A9* (p = 0.04). As previously described, individuals in the n-3 PUFA group displayed a lower systemic inflammatory response to LPS compared with placebo.[28] There was no change in calgranulin gene expression from n-3 PUFA vs. placebo supplementation prior to LPS administration in either the n-3 PUFA or placebo groups, indicating that the effect of n-3 PUFA on expression of these genes may be dependent on an additional immune stimulus.

### Expression of S100A8 and S100A9 is Higher in Adipose Tissue of Individuals with High Habitual n-3 PUFA Consumption

We hypothesized that this n-3 PUFA and inflammation-dependent difference in calgranulin expression may be more pronounced in a disease setting. In a cross-sectional study of obese metabolic syndrome patients (Gene Expression Omnibus (GEO) dataset GSE28070 analyzed using GEO2R)[43] we observed a significant difference in *S100A8* and *S100A9* expression in adipose tissue from individuals with low habitual n-3 PUFA intake (<1g/day, n = 6) vs. high habitual intake (>2g/day, n = 6). There was ~4-fold higher expression in individuals with n-3 PUFA intake >2g/day compared with those consuming <1g/day (*S100A8*, logFC = 1.92, adj p = 0.038; *S100A9*, logFC = 2.49, p = 0.027) consistent with a role for n-3 PUFA in enhancing expression in adipose tissue in the chronic inflammatory state of obesity. There were no significant differences between the groups in other known inflammatory genes, e.g. *IL6*, *IL10*, *CX3CL1*, *CCL2*, (all adj. p>0.1, logFC<1) indicating that n-3 PUFA modulated *S100A8* and *S100A9* specifically, rather than broadly modifying the overall inflammatory state.

### Tissue-specific Calgranulin Expression Response to LPS

**Fig 2:**
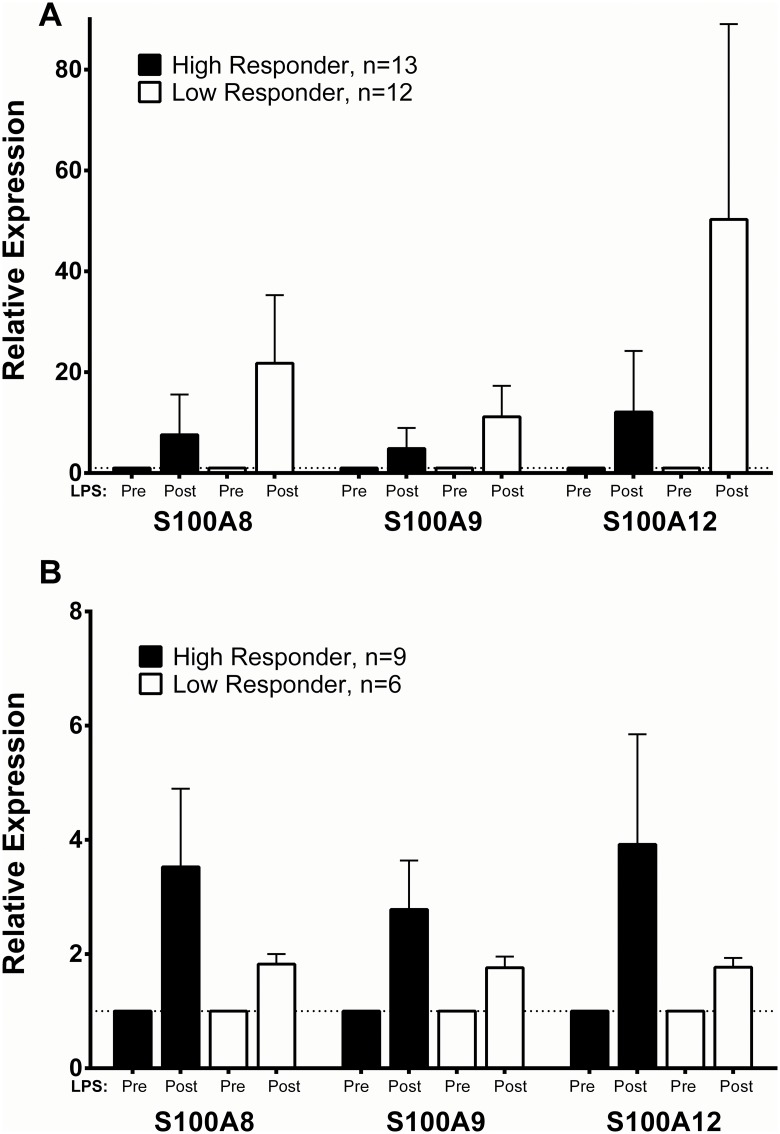
Up-regulation of *S100A8*, *S100A9* and *S100A12* in adipose tissue and CD14+ monocytes post-LPS in the GENE Study corresponds to the degree of systemic response to LPS, with a negative relationship in adipose tissue (A) and a positive relationship in CD14+ monocytes (B). Data plotted as mean fold change and standard deviation.

Our discovery sample was focused on adipose tissue. However, we hypothesized that there would also be an increase in gene expression in circulating mononuclear cells post-LPS *in vivo*. In an independent human endotoxemia protocol, the GENE Study,[30, 32] which administered a higher dose of LPS than the FFAME Study (1ng/kg LPS), we examined expression of *S100A8*, *S100A9* and *S100A12* pre- and post-LPS in adipose tissue (n = 25), and CD14+ monocytes (n = 15) of healthy individuals (n = 11 overlapped both tissues). There was a significant increase in all three genes in adipose tissue post-LPS: *S100A8* (15-fold, p = 0.001), *S100A9* (~10-fold, p = 0.001), and *S100A12* (~40-fold, p = 0.001). Further, expression of all three genes was increased significantly 2 hours post-LPS in CD14+ monocytes (*S100A8* 5-fold, p = 0.01; *S100A9* 4-fold, p = 0.01; *S100A12* 5-fold, p = 0.01). Because these individuals had previously been chosen for RNASeq based on their extreme phenotypic response to LPS (top and bottom 5% of the distribution for plasma cytokine and systemic febrile response), we examined the stratified gene expression changes in these “high” or “low” responders to LPS. We hypothesized that calgranulin gene expression in response to LPS would differ in individuals with strongly divergent clinical responses to LPS. Indeed, in adipose tissue, there was an approximately 3-fold greater increase in calgranulin gene up-regulation in “low” responders vs. “high” responders (Fig 2A). This corresponds to the findings in FFAME, where higher adipose expression in the n-3 PUFA group was associated with lower levels of systemic inflammatory activation. Conversely, in CD14+ monocytes, this directionality was reversed, with 2-3-fold higher up-regulation of *S100A8*, *S100A9* and *S100A12* in “high” responders compared with “low” responders. (Fig 2B), suggesting tissue-specific differences in response and function. To determine whether there was a relationship between calgranulin expression across tissues within the same individual, we examined correlations between adipose and CD14+ monocyte expression in the 11 individuals with expression data in both tissues. As expected, at each timepoint expression of all 3 genes correlated strongly and significantly with each other (e.g. pre-LPS CD14+ monocyte *S100A8* and *S100A9*, Spearman’s rho = 0.845, p<0.001). In CD14+ monocytes, baseline *S100A8* expression was positively correlated with post-LPS *S100A8* expression (Spearman’s rho = 0.74, P = 0.01). Strikingly, baseline CD14+ monocyte *S100A8* and *S100A9* expression was significantly negatively correlated with post-LPS adipose expression of all 3 genes, e.g. for *S100A9* baseline in CD14+ monocytes, the relationship with post-LPS adipose expression was as follows: *S100A8* Spearman’s rho = -0.8, P = 0.003; *S100A9* Spearman’s rho = -0.75, P = 0.007; *S100A12* Spearman’s rho = -0.67, P = 0.02). Thus, high calgranulin expression in circulating cells at baseline may predict low adipose tissue calgranulin induction during inflammatory stress.

### S100A9 Protein is Increased in Plasma Post-LPS

**Fig 3:**
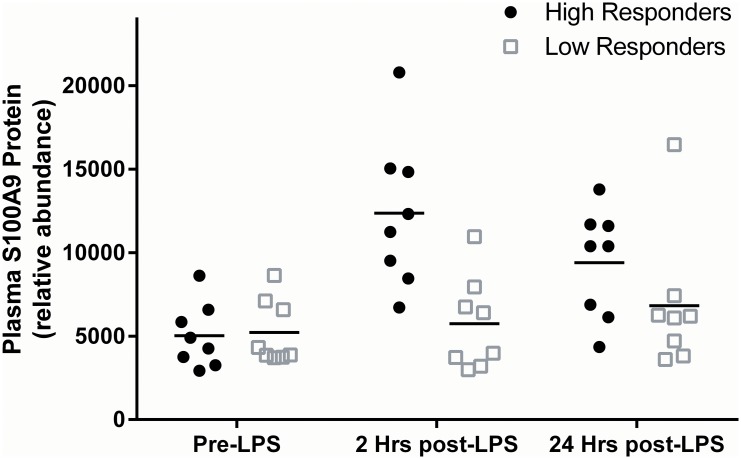
Plasma S100A9 protein following LPS in high and low systemic responders to evoked endotoxemia in the GENE Study. There was a significant difference in plasma S100A9 response to LPS between high and low responders (p = 0.01 for time*group interaction).

We probed whether the observed tissue increase in gene expression resulted in an increase in secreted protein levels. While plasma protein could not be measured in the FFAME Study (due to limited sample for secondary analyses), we measured plasma S100A9 levels in a subset of the individuals (n = 16) whose adipose and CD14+ monocyte gene expression was measured in the GENE Study.[30] S100A9 protein in plasma was increased significantly 2 hours post-LPS (1.8-fold, p = 0.01), and remained elevated 24-hours post-LPS (1.5-fold, p = 0.02). When stratified into “high” and “low” responders as described above, there was greater post-LPS increase in plasma S100A9 protein in high responders (n = 8) compared with low responders (n = 8), p = 0.01 for time*group interaction (Fig 3). Post-LPS levels of plasma S100A9 correlated significantly with post-LPS levels of IL-6 (Spearman’s rho = 0.9, P<0.0001), TNFα (Spearman’s rho = 0.8, P<0.001), and CXCL10 (Spearman’s rho = 0.8, P<0.0001). Interestingly, baseline levels of S100A9 correlated significantly with baseline CRP (Spearman’s rho = 0.6, P = 0.014). In contrast, plasma levels of IL6, TNFα and CXCL10 do not correlate with CRP. Thus, plasma S100A9 may be a marker both of acute inflammatory activation, and of chronic inflammatory stress.

### Expression of Calgranulin Genes is Modulated by n-3 PUFA Treatment and LPS in vitro, with Differential Effects in THP-1 Monocytes Compared to Adipocytes

**Fig 4:**
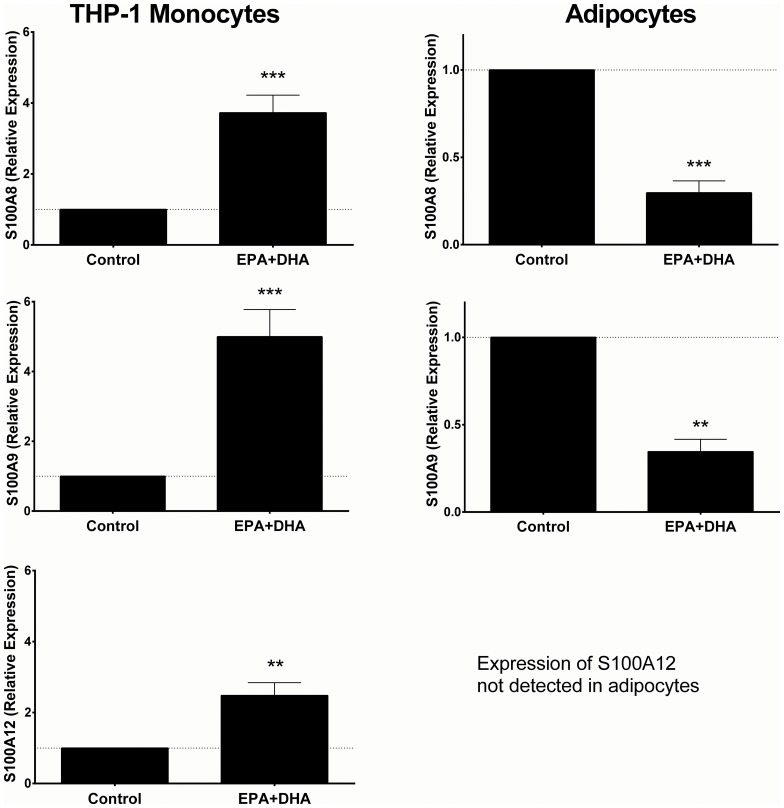
Expression of calgranulin genes (*S100A8*, *S100A9*, *S100A12*) is modulated by n-3 PUFA treatment in human THP-1 cells and adipocytes. *** indicates p<0.001; ** indicates p<0.01. Data plotted as mean fold change and standard deviation.

To further examine the divergent adipose and monocyte-specific responses, we examined cell-specific responses of calgranulin expression *in vitro* to n-3 PUFA and LPS in the THP-1 monocyte cell line and adipocytes. In THP-1 cells, expression of *S100A8*, *S100A9* and *S100A12* increased significantly following treatment with EPA + DHA (p<0.001 for *S100A8* and *S100A9*, p<0.01 for *S100A12*) (Fig 4). In contrast, in adipocytes, expression of both *S100A8* and *S100A9* decreased significantly with DHA + EPA treatment (p<0.001). We could not detect expression of *S100A12* in adipocytes, indicating that expression of this gene in adipose tissue may derive from other adipose cell sources (e.g. endothelial, macrophages, stromal cells).

**Fig 5:**
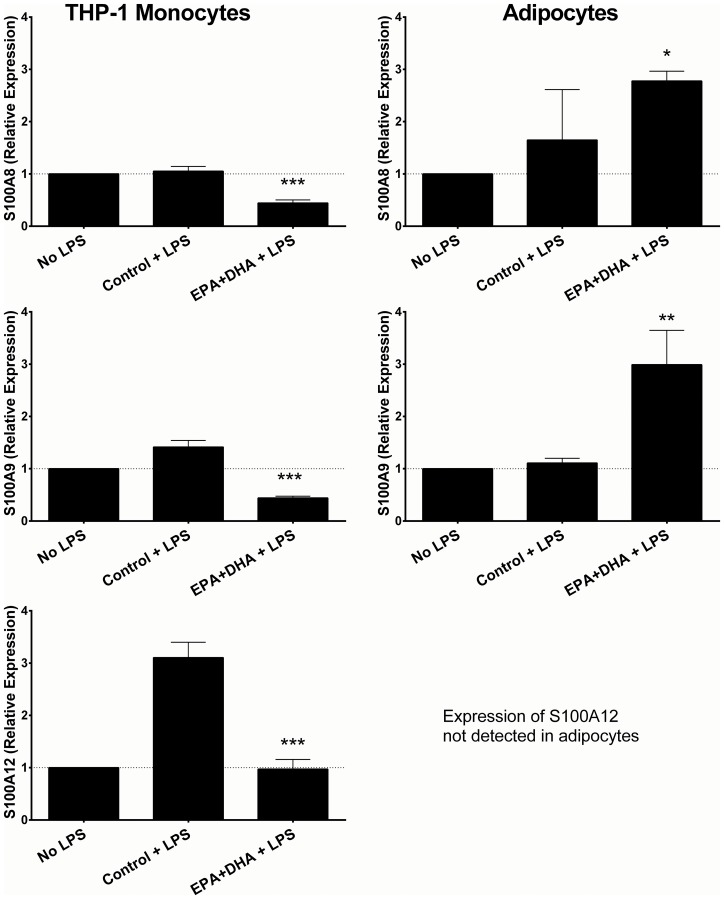
Expression of calgranulin genes (*S100A8*, *S100A9*, *S100A12*) in response to stimulation with LPS is modulated by n-3 PUFA treatment in human THP-1 cells and adipocytes. *** indicates p<0.001; ** indicates p<0.01; * indicates p<0.05. Data plotted as mean fold change and standard deviation.

In THP-1 cells, *S100A9* and *S100A12* were significantly up-regulated by LPS, while there was no significant change in *S100A8*. However, pre-treatment with n-3 PUFA prior to LPS resulted in significant attenuation of the LPS-induced increase in expression, with significantly lower expression following EPA+DHA treatment compared with control in all 3 genes (Fig 5). In adipocytes, while *S100A8* and *S100A9* were not significantly up-regulated by LPS alone, pre-treatment with EPA+DHA resulted in a significant LPS-induced increase in expression of both genes, again contrasting with the directionality of the effect in THP-1 cells, and consistent with the directionality of the *in vivo* data.

### Expression of Calgranulin Genes is Decreased During Maturation of Monocytes to Macrophages

We queried expression of calgranulins in freshly isolated monocytes and during maturation to macrophages (GEO data series GSE5099).[44] Expression of *S100A8* (LogFC = -4.09, p = 0.0003), *S100A9* (LogFC = -1.77, p = 0.005) and *S100A12* (LogFC = -4.85, p = 0.00005) decreased during maturation of monocytes to mature macrophages. In our own expression data from primary human macrophages and M1 or M2 polarized macrophages (available at GEO data series GSE55536),[37] there was a significant decrease in both *S100A8* (down ~3-fold, p = 0.004) and *S100A9* (down ~10-fold, p = 0.0004) during polarization to M1, and similarly during polarization to M2: *S100A8* down ~5-fold p = 0.005; S100A9 down ~3-fold p = 0.005, with a greater decrease in *S100A9* in M1 compared with M2 (p = 0.0004). There was no significant change in *S100A12* expression in these cells.

## Discussion

As important immune-mediating genes, calgranulins are of particular interest to chronic inflammatory diseases including cardiovascular disease. Dietary n-3 PUFA consumption may protect against a diverse range of immune-related diseases, although conflicting data on mechanisms and outcomes have hindered translational progress. Previous studies have examined the specific pro- or anti-inflammatory actions of *S100A8*, *S100A9* and *S100A12*, however to our knowledge these genes have not been studied for potential interaction with n-3 PUFA. We present evidence that consumption of n-3 PUFA is associated with enhanced expression of calgranulins within adipose tissue in response to an acute inflammatory challenge, as well as in the setting of chronic low-grade inflammation. We report novel differences in the response in adipocytes and adipose tissue compared with circulating mononuclear cells. Through integration of these data, we hypothesize that calgranulins mediatecell-specific n-3 PUFA-dependent regulation of systemic inflammation.

There was no difference in adipose calgranulin expression after n-3 PUFA treatment prior to endotoxemia in the FFAME study, consistent with the expectation that basal tissue inflammation in healthy individuals is at negligible levels. External endotoxemic inflammatory stress was required to observe expression differences in this lean and healthy sample. However in adipose tissue from obese individuals[43] there was higher expression in individuals with higher habitual intake (>2g/day) compared with low habitual intake (<1g/day). Given that obesity is associated with increased adipose inflammation,[45] it is possible that the increased inflammatory milieu in adipose from obese individuals modulates n-3 PUFA-associated difference, analogous to the evoked inflammatory challenge.

Strikingly, we observed tissue-specific differences in calgranulin gene expression in individuals with high or low systemic inflammatory responses (as assessed by fever and circulating cytokines). In adipose tissue *in vivo*, there was greater induction of calgranulins concurrent with lower systemic inflammatory response. This was observed in the FFAME Study, where the n-3 PUFA supplemented group had lower febrile and cytokine response to LPS than the placebo group. Similarly, in the GENE Study, where individuals were designated as “high” or “low” responders based on their febrile and cytokine response to LPS, the individuals with “low” response had higher post-LPS up-regulation of calgranulins in adipose tissue. Remarkably, within a subset of the same individuals in the GENE Study, “low” responders had lower post-LPS up-regulation of calgranulins in CD14+ monocytes. This striking difference raised the possibility of tissue-localization as crucially important in downstream consequences of calgranulin gene expression. The tissue-specific difference was recapitulated *in vitro*, where the effects of n-3 PUFA alone, as well as n-3 PUFA and LPS had opposing effects in adipocytes compared with THP-1 monocytes. Our data highlight that n-3 PUFA treatment has different effects on calgranulin expression in both adipocytes and monocytes when it is used alone, compared with the combination of n-3 PUFA and LPS. This could indicate that the relationship between n-3 PUFA and calgranulin expression is fundamentally altered during acute inflammatory stress compared with an unstressed state. Plasma responses of S100A9 protein were broadly consistent with CD14+ monocyte expression, indicating that plasma S100A9 protein may be primarily derived from secretion by circulating inflammatory cells rather than from adipose tissue.

Recent reports suggest that that *S100A8* and *S100A9* are involved in the recruitment of adipose tissue macrophages from bone marrow through a positive feedback loop,[18] and modulate leukocyte rolling and adhesion during recruitment.[46] *S100A8* and *S100A9* lead to inflammasome activation in a TLR4- and MYD88-dependent manner,[18] and stimulate CXCL10 production in monocytes and macrophages through TLR4 and NF-κB.[47] In our data, plasma S100A9 protein correlated significantly with plasma CXCL10 post-LPS (but not at baseline). In mice, *s100a8* adipose expression, as well as S100A8 protein was shown to stimulate macrophage migration as an early mediator of adipose inflammation in response to a pro-inflammatory obesogenic diet.[17]. In obese subjects, PBMC expression of *S100A8*, *S100A9* and *S100A12* correlated positively with visceral obesity.[48] Our data are consistent with a model where high circulating calgranulins are associated with increased chronic inflammation. Conversely, high adipose expression may be associated with reduced inflammation. Indeed, subjects with high baseline expression of calgranulins in CD14+ monocytes had lower induction of calgranulin expression in adipose tissue post-LPS. In the acute inflammatory setting, our novel findings suggest that the presence of LC n-3 PUFA may facilitate rapid up-regulation of calgranulin expression in adipose. This may lead to a more rapid resolution of systemic inflammation, potentially through an increase in macrophage recruitment and inflammasome activation. While intriguing, the correlative associations in our data do not demonstrate causality, and the mechanisms remains to be determined in future studies.

Our study has a number of strengths, but also some limitations. Our *in vivo* adipose tissue and CD14+ monocyte expression data reflects expression from a mixed cell population. *In vitro* expression data indicate higher *S100A8*, *S100A9* and *S100A12* expression in monocytes compared with mature macrophages, with further decreases during polarization to M1 or M2 macrophages. Thus, observed differences in expression within adipose tissue and in the circulation may relate to the proportion of monocytes compared with activated macrophages, rather than the number or activity of resident and infiltrating cells. The relative contribution of macrophage recruitment vs. macrophage activation status remains to be determined. Circulating or adipose tissue protein could not be measured in the FFAME Study due to limited sample availability, and thus the degree to which gene expression relates to translated protein could not be verified. However in the GENE Study expression of *S100A9* in adipose tissue post-LPS was significantly correlated with plasma protein. The specific functions of the calgranulin genes and interaction with n-3 PUFA may depend on formation of multimers, as well as intracellular vs. extracellular localization, which our data do not resolve. Given previous studies showing direct interaction between calgranulins and fatty acids[24, 25, 27], it is possible that the n-3 PUFA effects are mediated through direct binding or direct effects on stability of tertiary structure, however it remains possible that the effects are in fact mediated through fatty acid metabolites or alterations in membrane composition.

Given the increasing prevalence of complex inflammatory cardiometabolic diseases, strategies aimed at understanding specific mechanisms of disease development, and interaction with environmental factors, are of particular importance. We used a combined model of n-3 PUFA supplementation and evoked endotoxemia in healthy volunteers to understand the interplay between nutritional status and inflammatory responses prior to development of cardiometabolic disease. Through *in vivo* and *in vitro* interrogation, we highlight calgranulins *S100A8*, *S100A9* and *S100A12* as n-3 PUFA-dependent regulators of systemic and adipose tissue inflammatory responses, and highlight a potential novel mechanism for n-3 PUFA modulation of inflammation.

## Supporting Information

S1 File Data used to prepare Figs 4 and 5 is presented in S1_File. (XLSX)

## Abbreviations

DHA: Docosahexaenoic Acid
EPA: Eicosapentaenoic Acid
FFAME Study: Fenofibrate and omega-3 fatty acid modulation of endotoxemia Study
FPKM: Fragments per kilobase per million fragments mapped
LC n-3 PUFA: long-chain omega 3 polyunsaturated fatty acid
LPS: Lipopolysaccharide
RNASeq: RNA Sequencing
